# Supplementary material for: Gene editing and scalable functional genomic screening in Leishmania species using the CRISPR/Cas9 cytosine base editor toolbox LeishBASEedit
Source: eLife. 2023 May 24;12:e85605. doi: 10.7554/eLife.85605 (PMC10208639; doi:10.7554/eLife.85605)
Supplement: Supplementary file 2. — L. donovani, L. infantum, L. mexicana, and L. major wildtype parasites were transfected with pLdCH-hyBE4max-sgRNA expression plasmids, targeting PF16, MFT, PFR2, and IFT88 with four guides each (see main text description). Trace plots before transfection (control sample) and 28 days after transfection (edited sample) were aligned using ICE (Syntheco). Horizontal black line: 20nt guide target sequence. Horizontal red dotted line: PAM sequence. Vertical grey dotted line: nCas9 (D10A) DNA single-strand break (nick) position. [file elife-85605-supp2.pdf]

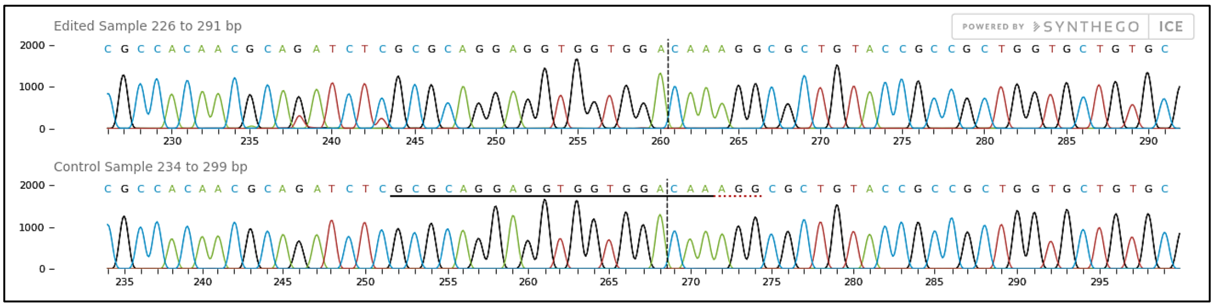


**pLdCH-hyBE4max-PF16-1 (GCGCAGGAGGTGGTGGACAA) in *L. donovani* (LdBPK_201450.1)**

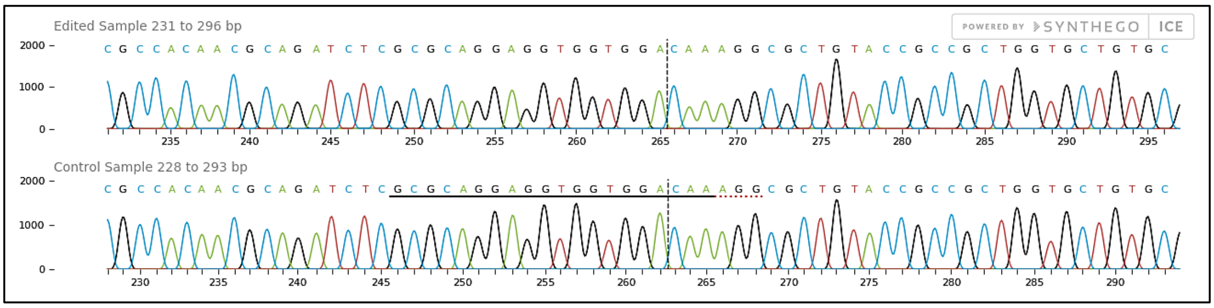


**pLdCH-hyBE4max-PF16-1 (GCGCAGGAGGTGGTGGACAA) in *L. infantum* (LINF_200019300)**

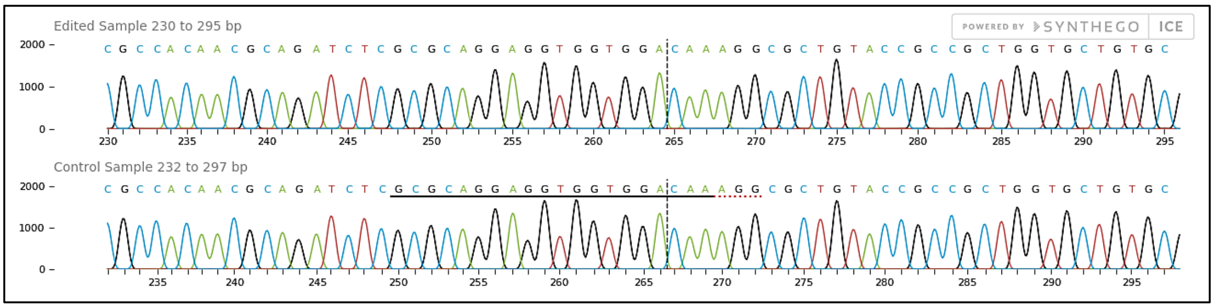


**pLdCH-hyBE4max-PF16-1 (GCGCAGGAGGTGGTGGACAA) in *L. major* (LmjF.20.1400)**

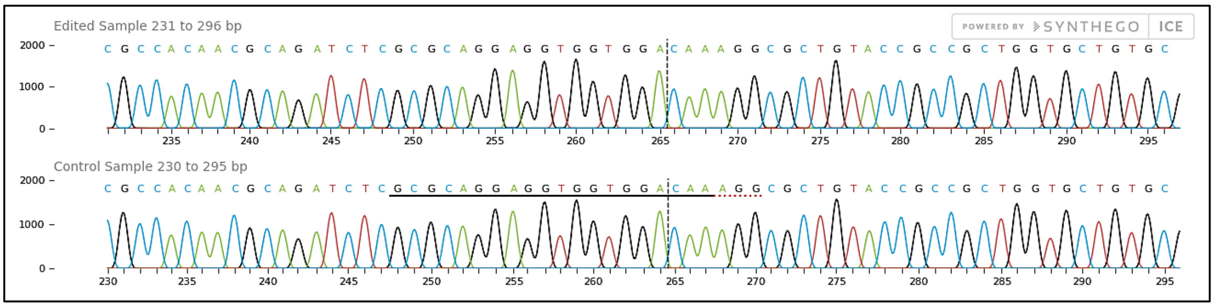


**pLdCH-hyBE4max-PF16-1 (GCGCAGGAGGTGGTGGACAA) in *L. mexicana* (LmxM.20.1400)**

**Figure S9**

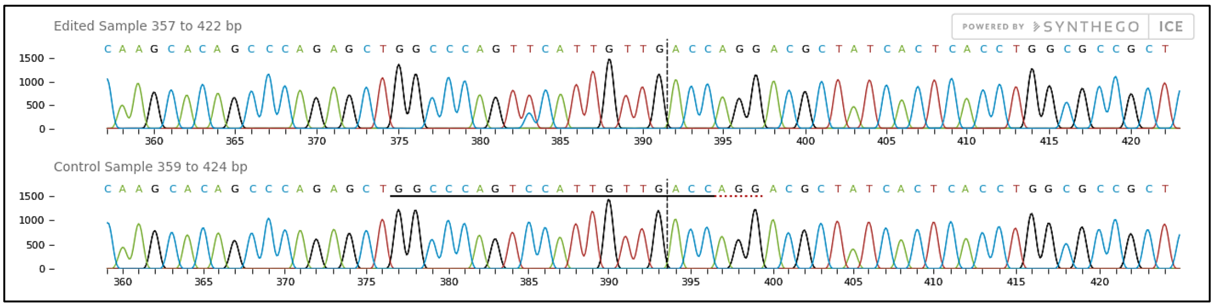


**pLdCH-hyBE4max-PF16-2 (GGCCCAGTCCATTGTTGACC) in *L. donovani* (LdBPK_201450.1)**

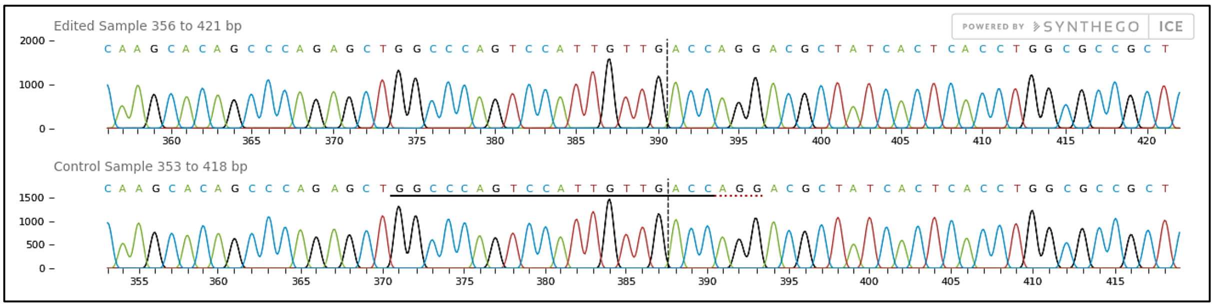


**pLdCH-hyBE4max-PF16-2 (GGCCCAGTCCATTGTTGACC) in *L. infantum* (LINF_200019300)**

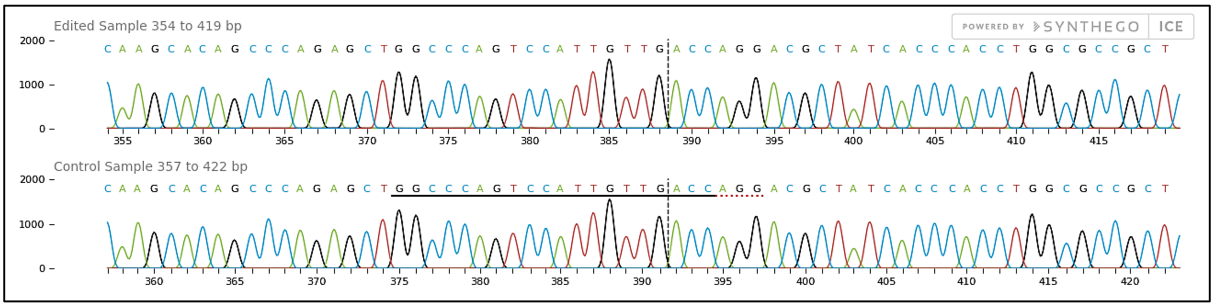


**pLdCH-hyBE4max-PF16-2 (GGCCCAGTCCATTGTTGACC) in *L. major* (LmjF.20.1400)**

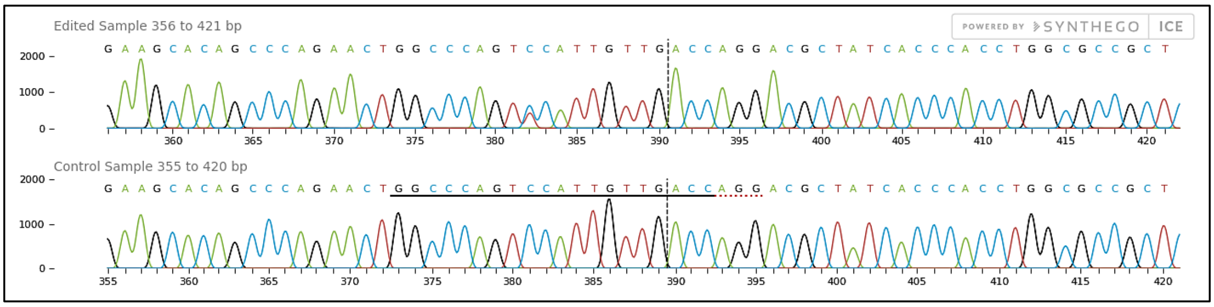


**pLdCH-hyBE4max-PF16-2 (GGCCCAGTCCATTGTTGACC) in *L. mexicana* (LmxM.20.1400)**

**Figure S9**

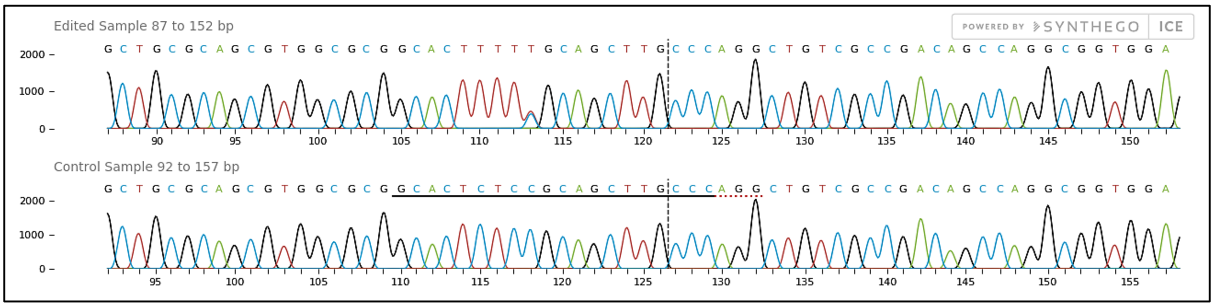


**pLdCH-hyBE4max-PF16-3 (GCACTCTCCGCAGCTTGCCC) in *L. donovani* (LdBPK_201450.1)**

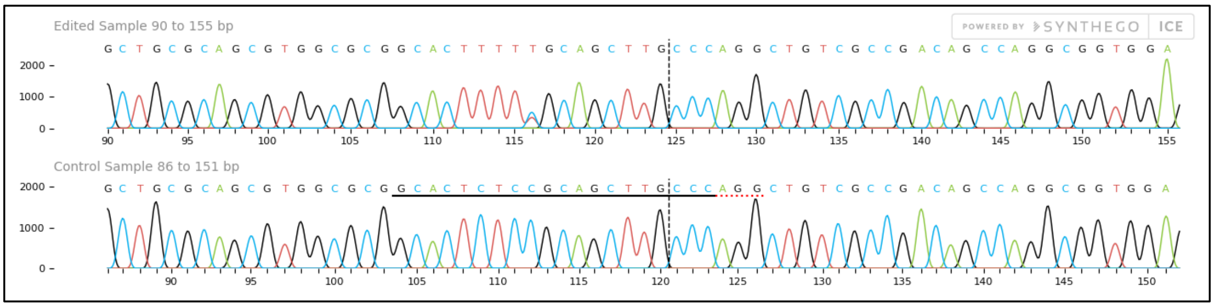


**pLdCH-hyBE4max-PF16-3 (GCACTCTCCGCAGCTTGCCC) in *L. infantum* (LINF_200019300)**

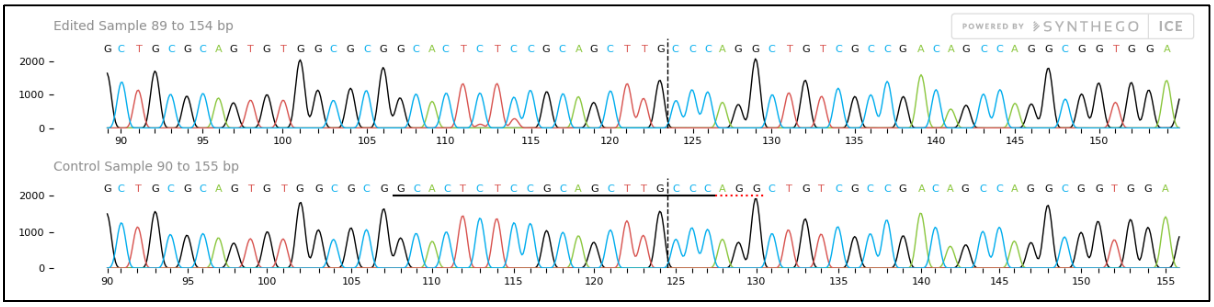


**pLdCH-hyBE4max-PF16-3 (GCACTCTCCGCAGCTTGCCC) in *L. major* (LmjF.20.1400)**

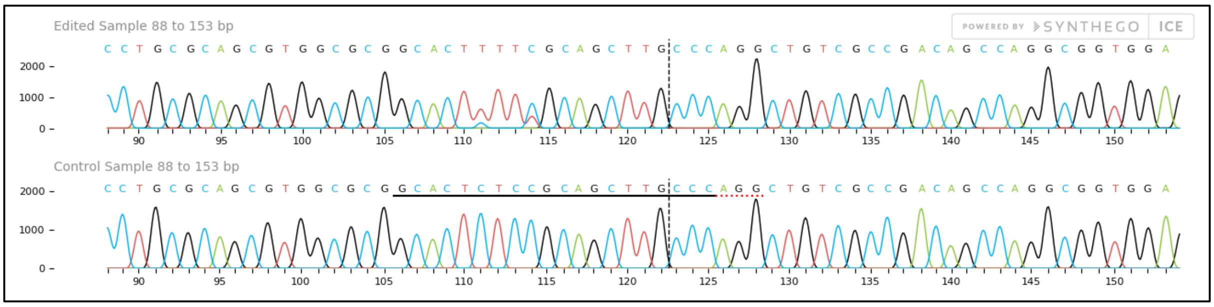


**pLdCH-hyBE4max-PF16-3 (GCACTCTCCGCAGCTTGCCC) in *L. mexicana* (LmxM.20.1400)**

**Figure S9**

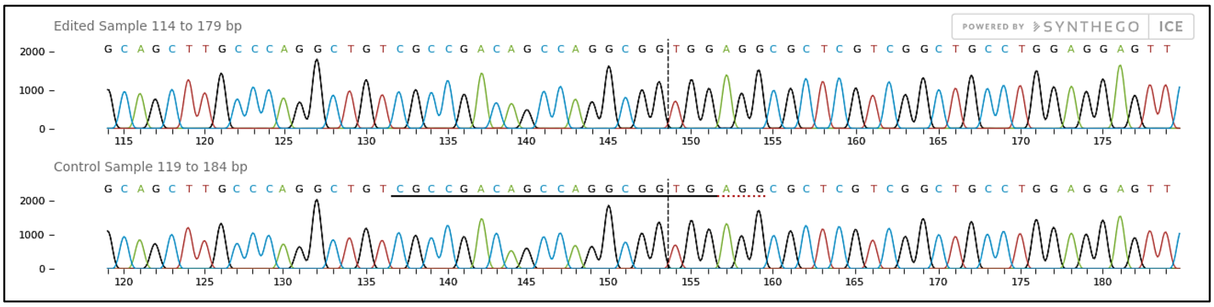


**pLdCH-hyBE4max-PF16-4 (CGCCGACAGCCAGGCGGTGG) in *L. donovani* (LdBPK_201450.1)**

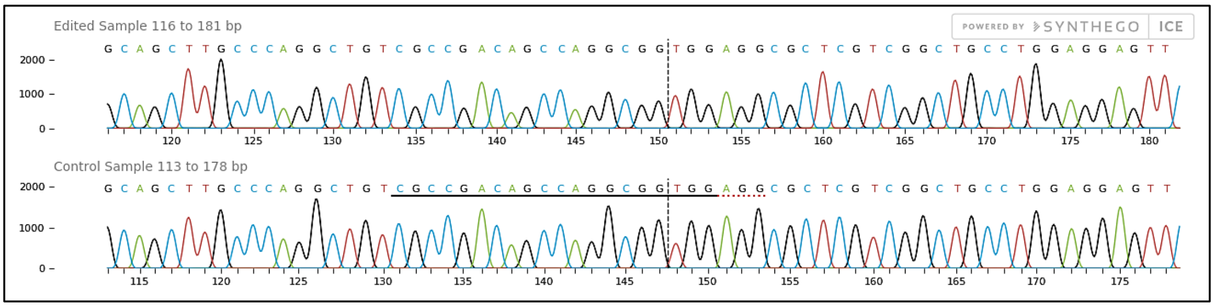


**pLdCH-hyBE4max-PF16-4 (CGCCGACAGCCAGGCGGTGG) in *L. infantum* (LINF_200019300)**

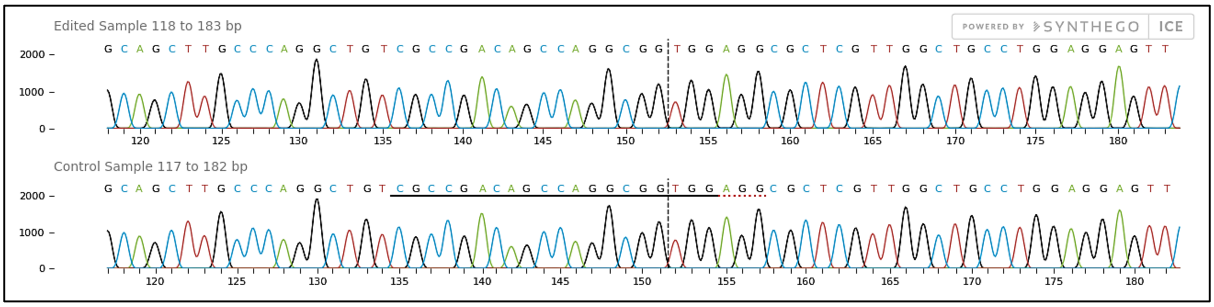


**pLdCH-hyBE4max-PF16-4 (CGCCGACAGCCAGGCGGTGG) in *L. major* (LmjF.20.1400)**

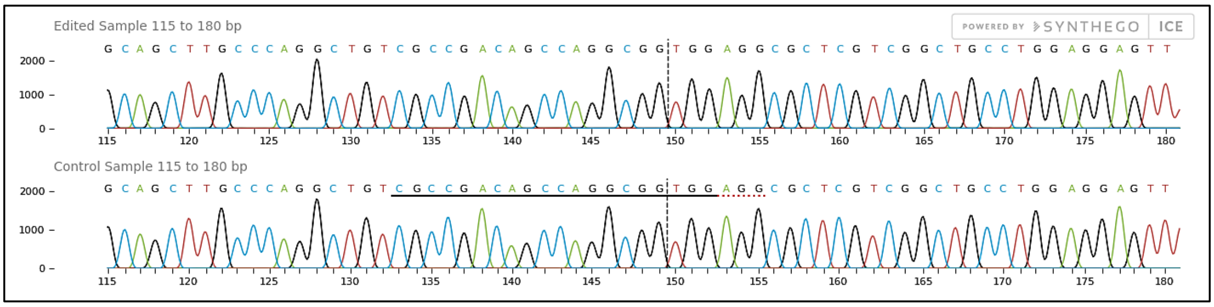


**pLdCH-hyBE4max-PF16-4 (CGCCGACAGCCAGGCGGTGG) in *L. mexicana* (LmxM.20.1400)**

**Figure S9**

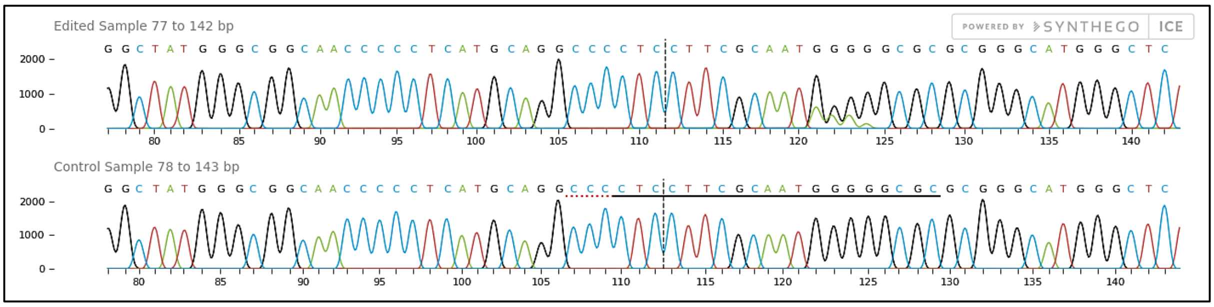


**pLdCH-hyBE4max-IFT88-1 (GCGCCCCCATTGCGAAGGAG) in *L. infantum* (LINF_270017700)**

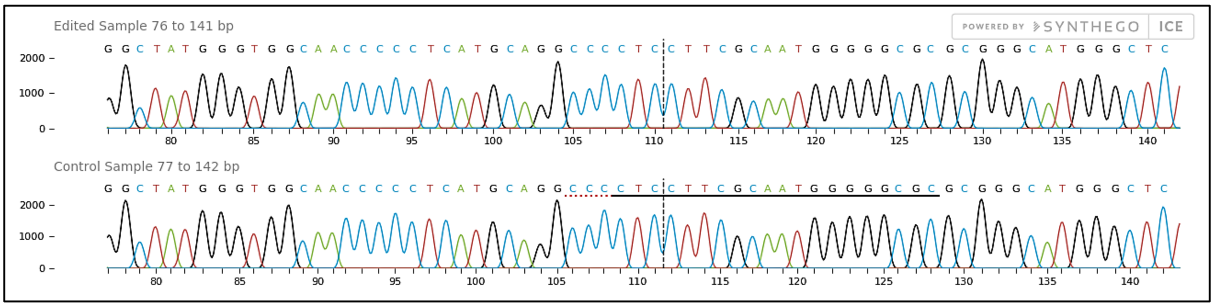


**pLdCH-hyBE4max-IFT88-1 (GCGCCCCCATTGCGAAGGAG) in *L. major* (LmjF.27.1130)**

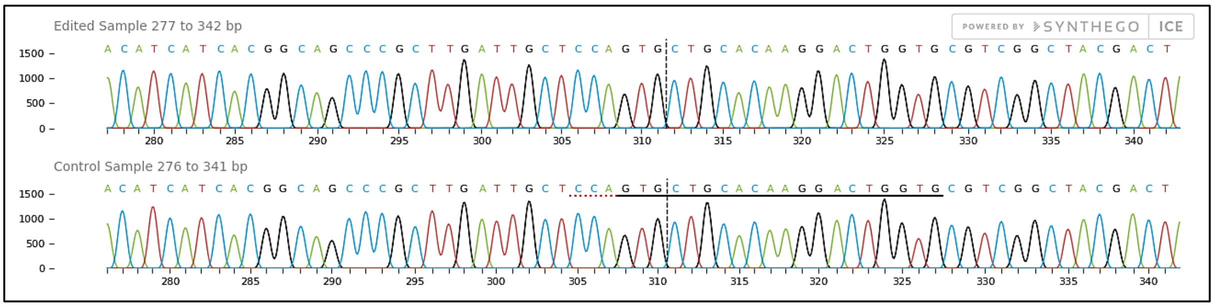


**pLdCH-hyBE4max-IFT88-2 (CACCAGTCCTTGTGCAGCAC) in *L. infantum* (LINF_270017700)**

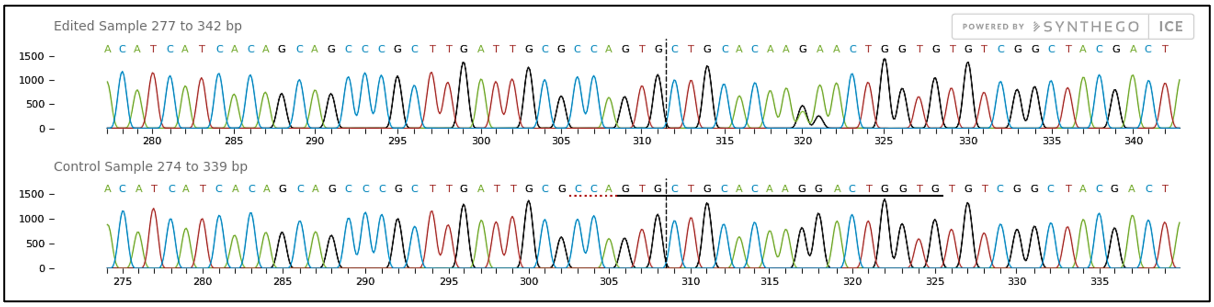


**pLdCH-hyBE4max-IFT88-2 (CACCAGTCCTTGTGCAGCAC) in *L. major* (LmjF.27.1130)**

**Figure S9**

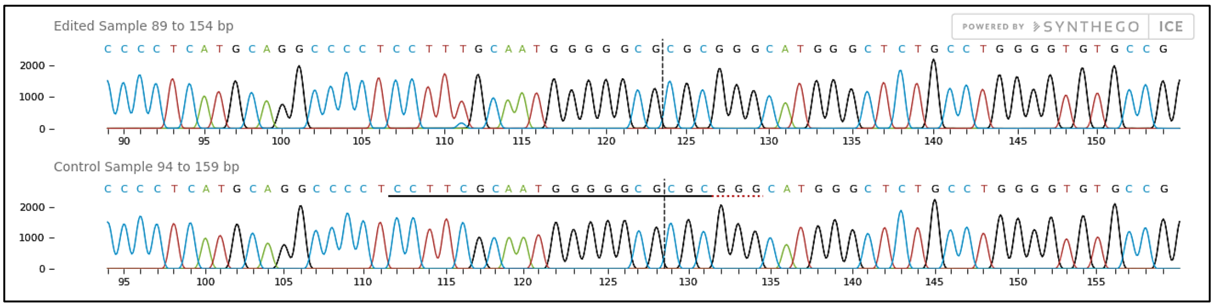


**pLdCH-hyBE4max-IFT88-3 (CCTTCGCAATGGGGGCGCGC) in *L. infantum* (LINF_270017700)**

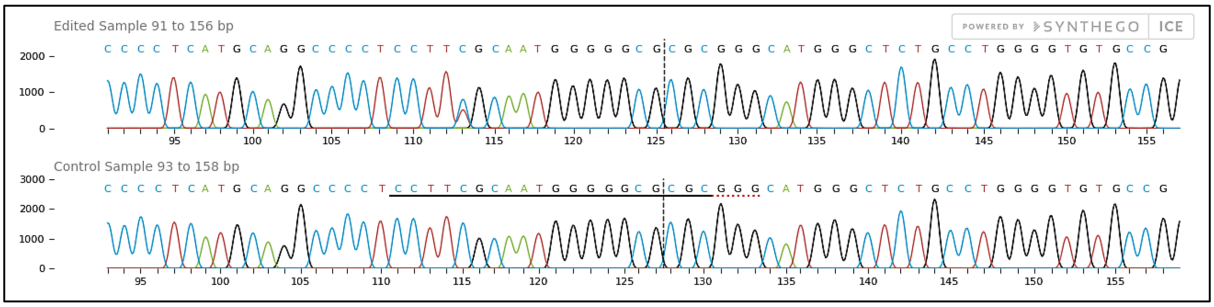


**pLdCH-hyBE4max-IFT88-3 (CCTTCGCAATGGGGGCGCGC) in *L. major* (LmjF.27.1130)**

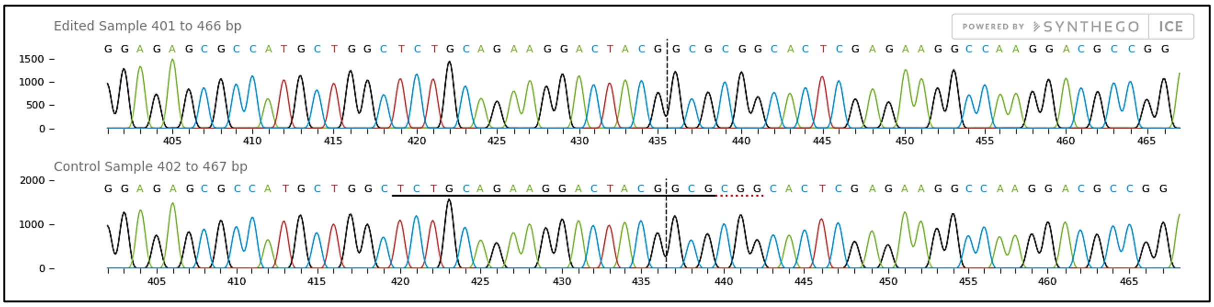


**pLdCH-hyBE4max-IFT88-4 (TCTGCAGAAGGACTACGGCG) in *L. infantum* (LINF_270017700)**

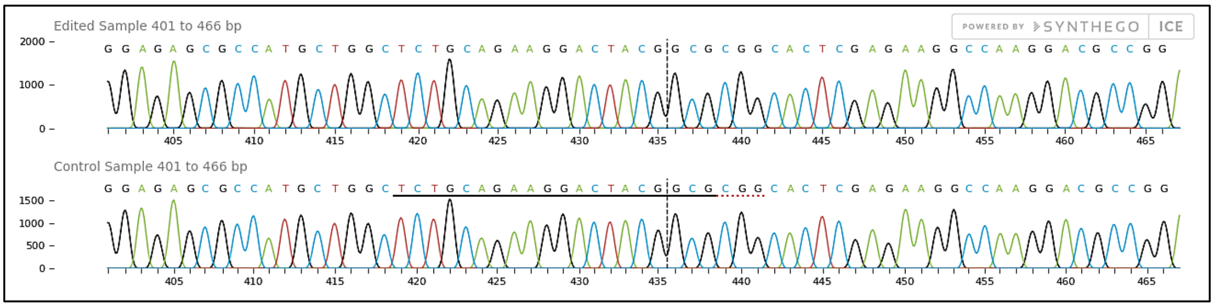


**pLdCH-hyBE4max-IFT88-4 (TCTGCAGAAGGACTACGGCG) in *L. major* (LmjF.27.1130)**

**Figure S9**

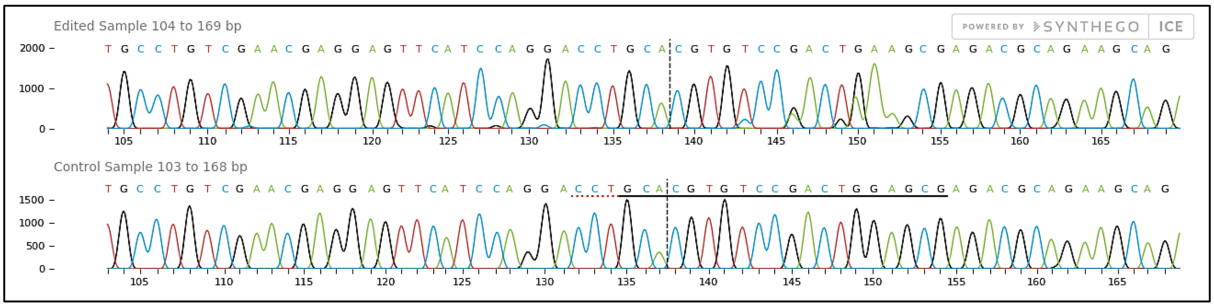


**pLdCH-hyBE4max-PFR2-1 (CGCTCCAGTCGGACACGTGC) in *L. mexicana* (LmxM.16.1430.1)**

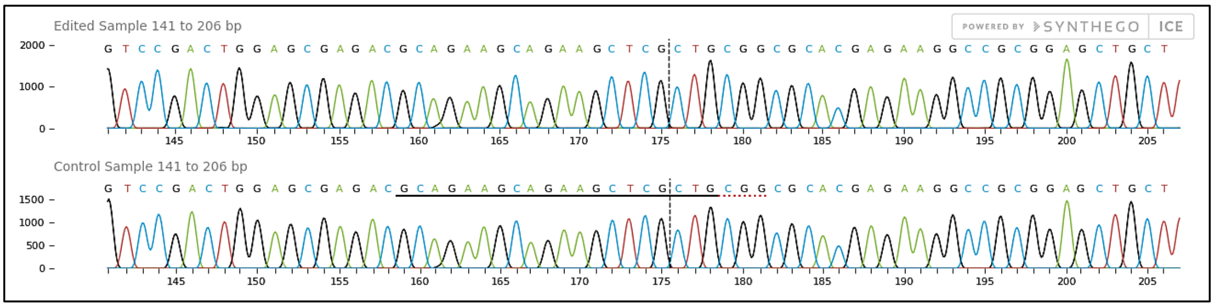


**pLdCH-hyBE4max-PFR2-2 (GCAGAAGCAGAAGCTCGCTG) in *L. mexicana* (LmxM.16.1430.1)**

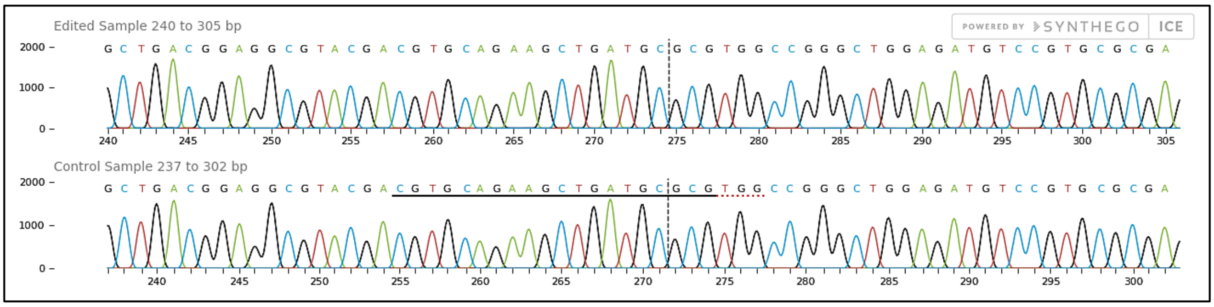


**pLdCH-hyBE4max-PFR2-3 (CGTGCAGAAGCTGATGCGCG) in *L. mexicana* (LmxM.16.1430.1)**

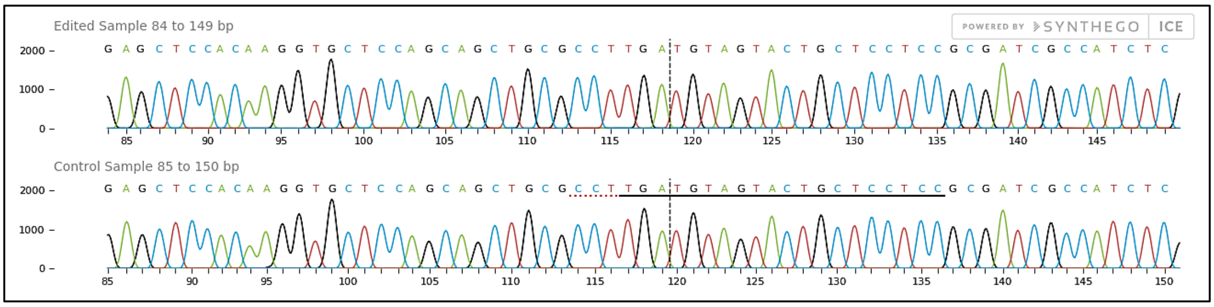


**pLdCH-hyBE4max-PFR2-4 (GGAGGAGCAGTACTACATCA) in *L. mexicana* (LmxM.16.1430.1)**

**Figure S9**

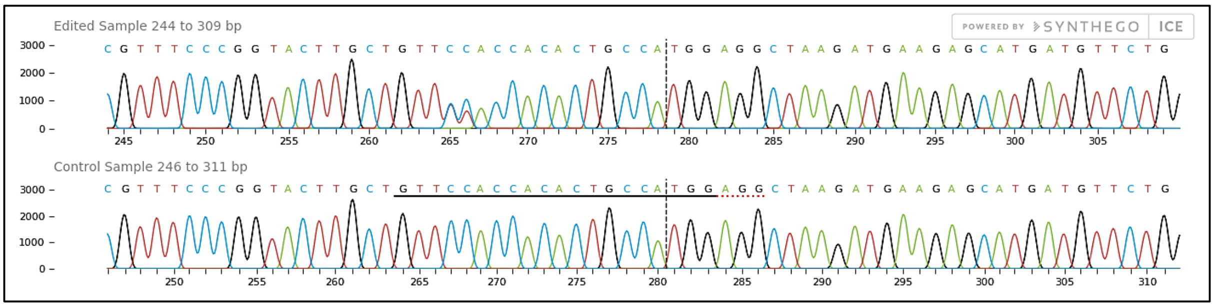


**pLdCH-hyBE4max-MFT-1 (GTTCCACCACACTGCCATGG)** ***L. donovani*** **(LdBPK_131590.1) <u>Not</u> pre-treated**

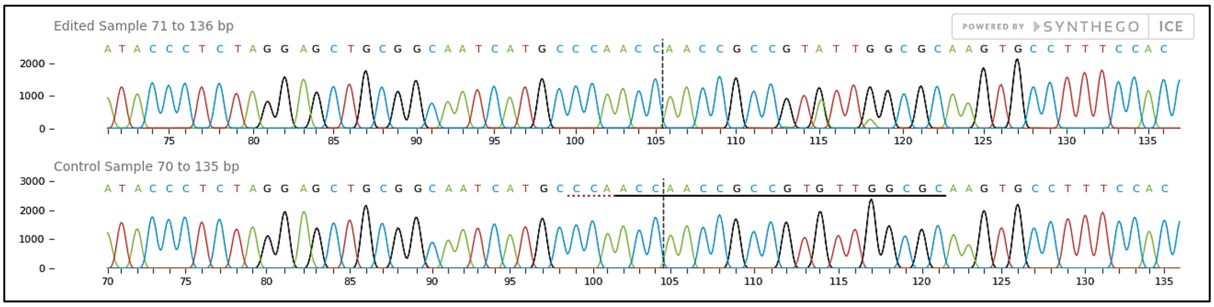


**pLdCH-hyBE4max-MFT-2 (GCGCCAACACGGCGGTTGGT)** ***L. donovani*** **(LdBPK_131590.1) <u>Not</u> pre-treated**

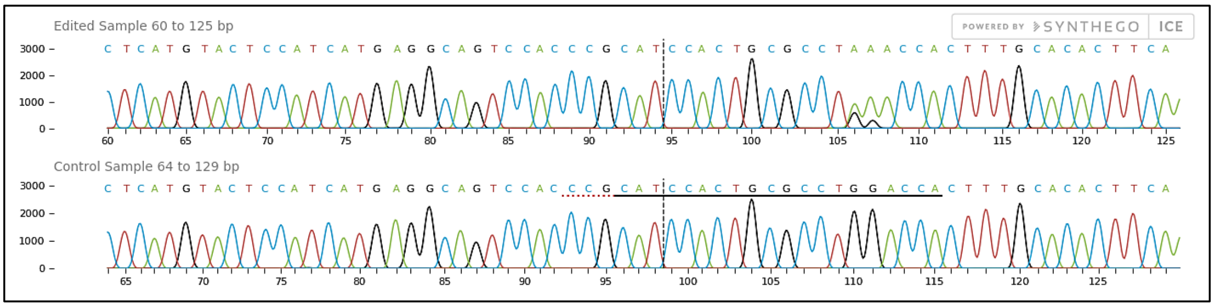


**pLdCH-hyBE4max-MFT-3 (TGGTCCAGGCGCAGTGGATG)** ***L. donovani*** **(LdBPK_131590.1) <u>Not</u> pre-treated**

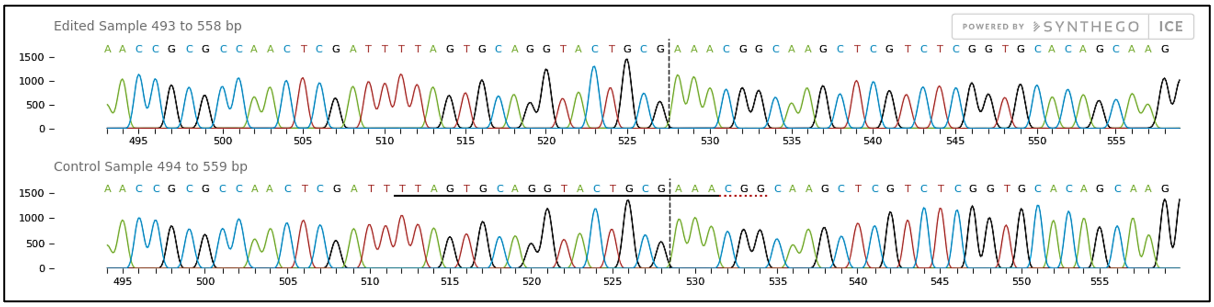


**pLdCH-hyBE4max-MFT-4 (TTAGTGCAGGTACTGCGAAA)** ***L. donovani*** **(LdBPK_131590.1) <u>Not</u> pre-treated**

**Figure S9**

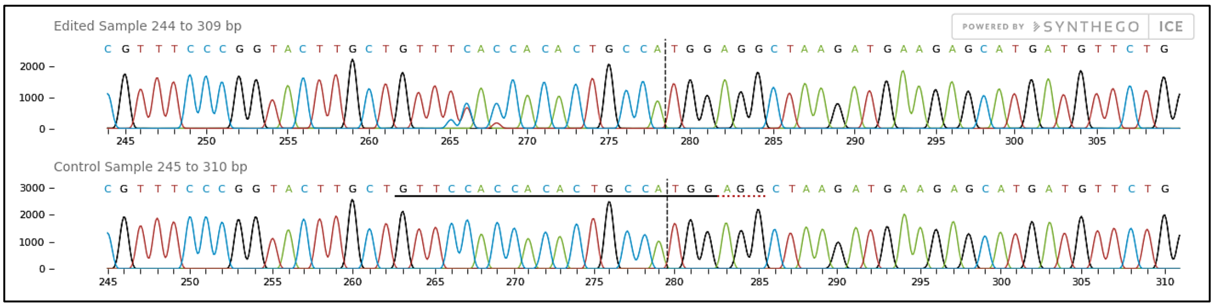


**pLdCH-hyBE4max-MFT-1 (GTTCCACCACACTGCCATGG) *L. donovani* (LdBPK_131590.1) pre-treated**

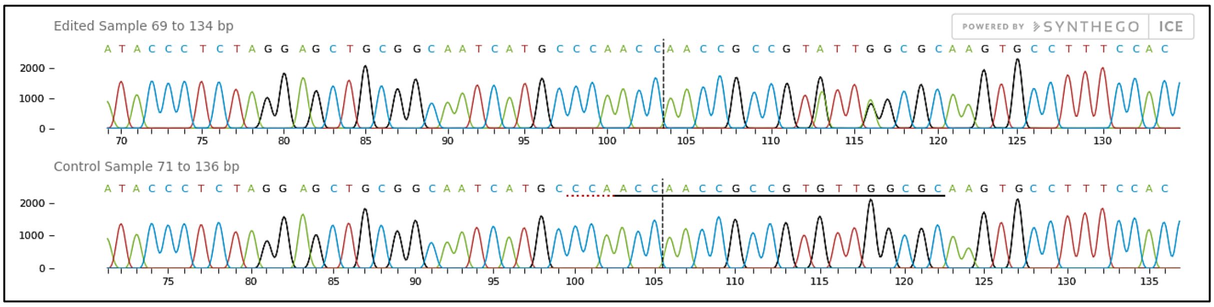


**pLdCH-hyBE4max-MFT-2 (GCGCCAACACGGCGGTTGGT) *L. donovani* (LdBPK_131590.1) pre-treated**

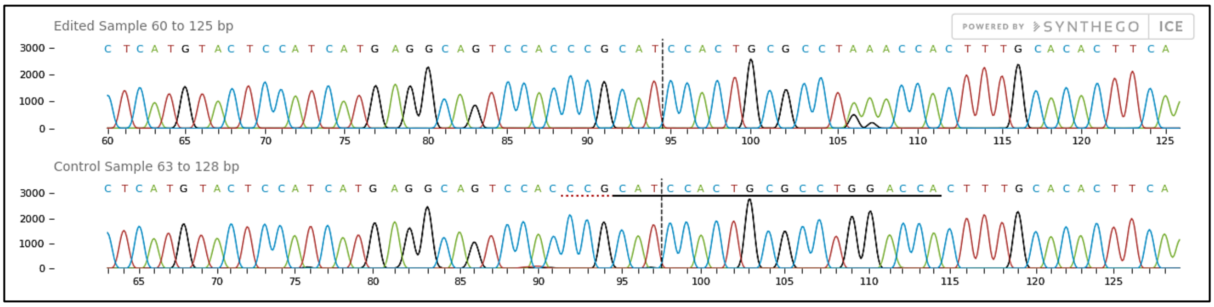


**pLdCH-hyBE4max-MFT-3 (TGGTCCAGGCGCAGTGGATG) *L. donovani* (LdBPK_131590.1) pre-treated**

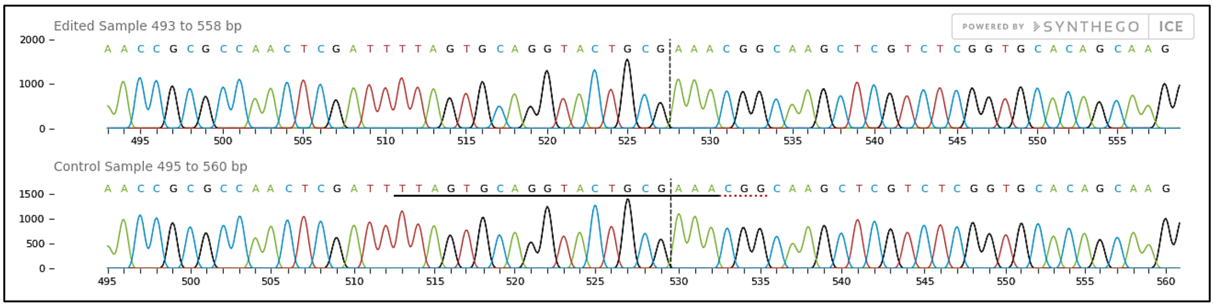


**pLdCH-hyBE4max-MFT-4 (TTAGTGCAGGTACTGCGAAA) *L. donovani* (LdBPK_131590.1) pre-treated**

## Figure S9
