# Supplementary material for: Gene editing and scalable functional genomic screening in Leishmania species using the CRISPR/Cas9 cytosine base editor toolbox LeishBASEedit
Source: eLife. 2023 May 24;12:e85605. doi: 10.7554/eLife.85605 (PMC10208639; doi:10.7554/eLife.85605)
Supplement: Supplementary file 6. — A step-by-step protocol to anneal oligos and clone sgRNAs into pLdCH-hyBE4max and pLdCH-hyBE4max-LmajDBD. [file elife-85605-supp6.pdf]

Julius-Maximilians-
UNIVERSITÄT
WÜRZBURG
:::::: Biozentrum
Zell- und Entwicklungsbiologie
.ZOO

LeishBASEedit

www.biozentrum.uni-wuerzburg.de/zeb/team/staff-scientists/tom-beneke/

Dr. Tom Beneke

Cell and Developmental Biology
Biocenter, University of Wuerzburg
Am Hubland • D-97074 Würzburg
tom.beneke@uni-wuerzburg.de

Cloning guide target sequences into base editor plasmids for *Leishmania* editing
Protocol version October 2022

Workflow

Primer pairs given by LeishBASEedit can be used to clone guides into BbsI sites of base editor plasmids, such as pLdCH-hyBE4max. The general workflow is shown below. First, both oligos (primer sequences given by LeishBASEedit primer design) are annealed and phosphorylated, before they are being ligated into a dephosphorylated vector that was digested with BbsI. The resulting plasmid can be transfected into *Leishmania* species.

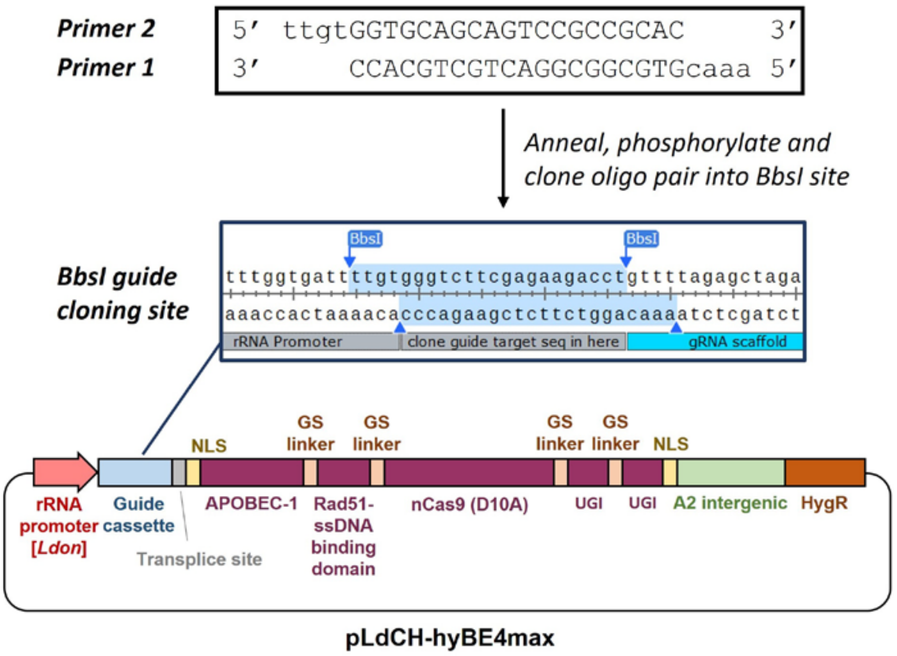

Step-by-step protocol

1. <u>Digest and Dephosphorylate base editor plasmid</u>
   - 5 µg plasmid
   - 2 µl BpiI (BbsI) [ThermoFisher, ER1011]
   - 2 µl FastAP [ThermoFisher, EF0651]
   - 3 µl 10x Tango Buffer [ThermoFisher]
   - 30 µl total volume with ddH_2O
   - Incubate at 37°C for at least 4 hours or better overnight
   - PCR purify digested and dephosphorylated plasmid (confirm linearization of plasmid on gel)

2. <u>Anneal and phosphorylate oligo pair</u>
   - Order standard desalted oligos (25 nmole scale)
   - 1 µl oligo 1 (100 µM)
   - 1 µl oligo 2 (100 µM)
   - 1 µl T4 DNA Ligation Buffer (ATP needed for phosphorylation)
   - 0.5 µl T4-Polynukleotid-Kinase [ThermoFisher, EK0031]
   - 6.5 µl ddH_2O
   - Phosphorylate at 37°C for 30 minutes, followed by 5 minutes at 95°C
   - Then anneal by ramping down from 95°C to 25°C at 5°C/minute
   - Dilute (1:200) annealed and phosphorylated oligo pair by mixing 1µl with 199µl ddH_2O

3. <u>Ligation and transformation</u>
   - 50ng digested and dephosphorylated plasmid (from Step 1)
   - 1µl diluted oligo pair (from Step 2)
   - 1µl T4 DNA Ligation Buffer
   - 0.5µl T4 DNA Ligase [ThermoFisher, EL0014]
   - 10µl total volume with ddH_2O
   - Incubate at 37°C for 2 hours
   - Transform entire volume into 50-100 µl competent cells (e.g. TOP10, Stbl3, XL1Blue)
   - Spread out on AmpR plate and compare to “no-insert” control
