# Supplementary material for: Gene editing and scalable functional genomic screening in Leishmania species using the CRISPR/Cas9 cytosine base editor toolbox LeishBASEedit
Source: eLife. 2023 May 24;12:e85605. doi: 10.7554/eLife.85605 (PMC10208639; doi:10.7554/eLife.85605)
Supplement: Supplementary file 8. — A step-by-step protocol to transfect pLdCH-hyBE4max and pLdCH-hyBE4max-LmajDBD guide expression plasmids into Leishmania parasites. [file elife-85605-supp8.pdf]

Julius-Maximilians-
UNIVERSITÄT
WÜRZBURG
:::::: Biozentrum
Zell- und Entwicklungsbiologie
.ZOO

www.biozentrum.uni-wuerzburg.de/zeb/team/staff-scientists/tom-beneke/

Dr. Tom Beneke

Cell and Developmental Biology
Biocenter, University of Wuerzburg
Am Hubland • D-97074 Würzburg
tom.beneke@uni-wuerzburg.de

Transfecting base editor guide plasmids into *Leishmania*
Protocol version October 2022

Step-by-step protocol

Base editor plasmids that contain guide target sequences can be transfected into *Leishmania* species following conditions outlined in Schumann Burkard et al., Mol Biochem Parasitol 2011; 175, 91-94.

Transfection Buffer

1. Prepare the following buffers:

**3x Tb-BSF buffer stock**	**$CaCl_2$ stock**
200 mM Na_2HPO_4	1.5 mM $CaCl_2$ in water
70 mM NaH_2PO_4	
15 mM KCl	
150 mM HEPES pH 7.4	

Cell-Plasmid-Transfection-Mix

1. Dilute 5-10 µg of plasmid DNA into 50 µl ddH_2O and heat sterilize for 5 minutes at 95°C
2. For each transfection (see transfection protocol below) a Cell-Transfection-Mix is prepared by mixing:
   - 25 µl $CaCl_2$
   - 83 µl 3x Tb-BSF
   - 92 µl ddH_2O and

Transfection protocol

1. Prepare an exponentially growing culture of *Leishmania*
2. Prepare the required volume of transfection buffer according to instructions above (ensure all solutions are sterile)
3. Pre-warm medium and collect the required number of cells (at least 5E6 cells per transfection) at 800g for 5 minutes
4. Take off supernatant and wash in 1 – 5 ml of Cell-Transfection-Mix (as specified above)
5. Resuspend pellet in desired final volume of Cell-Transfection-Mix
6. Mix 200 µl Cell-Transfection-Mix with 50 µl heat sterilized plasmid DNA and transfer into electroporation cuvette. (Note: Leave the cells in the cuvettes for the shortest possible time. When doing a big batch of transfections, do no more than five cuvettes at a time and keep the remaining cells in the Eppendorf tube until use)
7. Transfection: one pulse with X-001 (Amaxa Nucleofector 2b)
8. Quickly and carefully transfer cells into 5 ml pre-warmed medium (cell concentration should not be higher than 1E6 cells/ml) and rinse cuvette once with medium.
9. Leave cells to incubate for 8-16 h, then add the required selection drug and incubate until drug resistant populations emerge
