# Supplementary figures and images for: Gene editing and scalable functional genomic screening in Leishmania species using the CRISPR/Cas9 cytosine base editor toolbox LeishBASEedit
Source: eLife. 2023 May 24;12:e85605. doi: 10.7554/eLife.85605 (PMC10208639; doi:10.7554/eLife.85605)

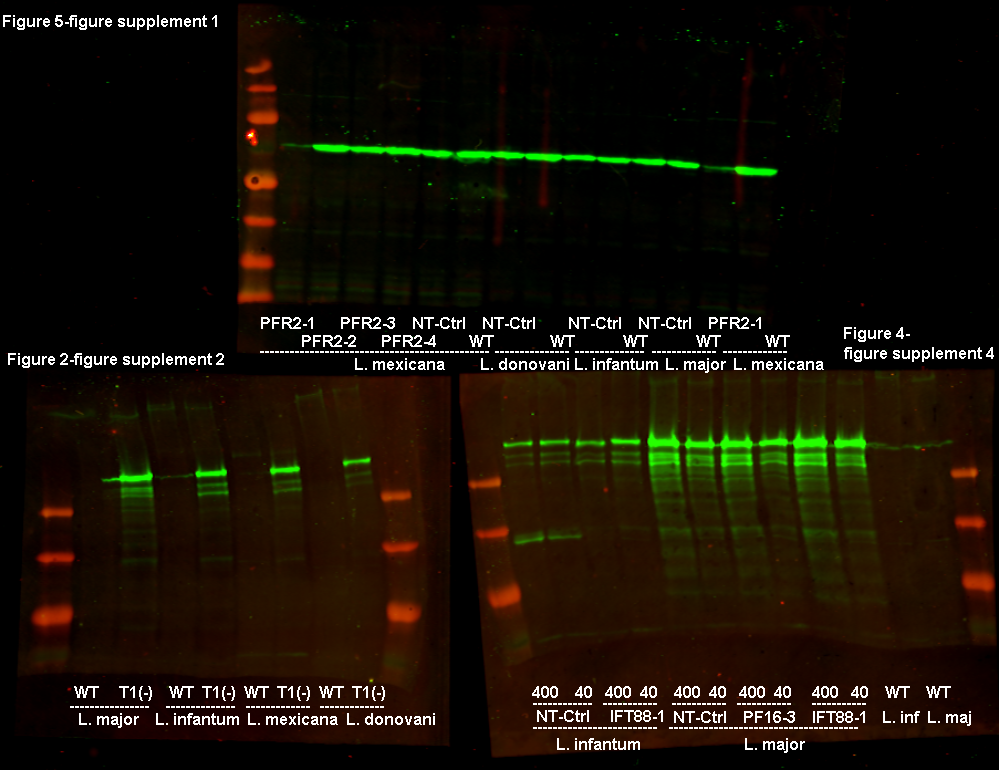

Supplement: Source data 2. — Nitrocellulose membranes with Leishmania promastigote protein samples were subject to western blots and imaged using a LI-COR Odyssey CLx. This is the raw TIF file of this scan. File “Source data 2 (labels)” is the same file but with identities of protein samples highlighted. Details for samples can be found in legends of Figure 2-figure supplement 2 (bottom-left panel), Figure 4-figure supplement 4 (bottom-right panel) and Figure 5-figure supplement 1 (top panel). [file elife-85605-data2.zip › Figure 2-figure supplement 2, Figure 4-figure supplement 4, Figure 5-figure supplement 1 - source data 2 (labels).tif]

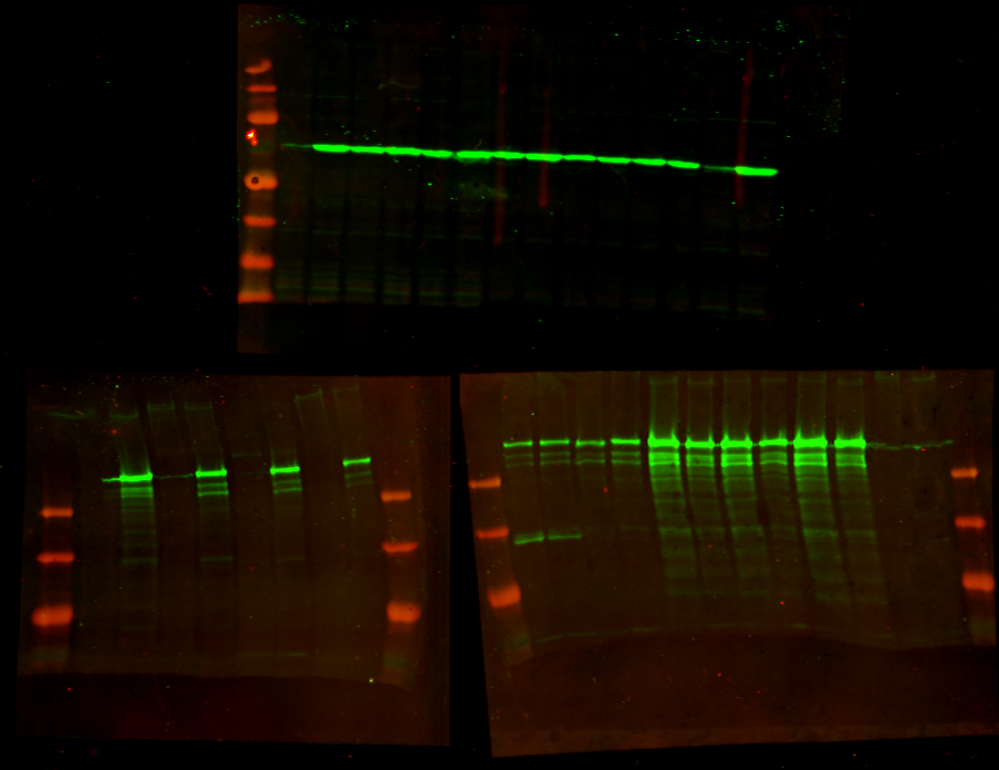

Supplement: Source data 2. — Nitrocellulose membranes with Leishmania promastigote protein samples were subject to western blots and imaged using a LI-COR Odyssey CLx. This is the raw TIF file of this scan. File “Source data 2 (labels)” is the same file but with identities of protein samples highlighted. Details for samples can be found in legends of Figure 2-figure supplement 2 (bottom-left panel), Figure 4-figure supplement 4 (bottom-right panel) and Figure 5-figure supplement 1 (top panel). [file elife-85605-data2.zip › Figure 2-figure supplement 2, Figure 4-figure supplement 4, Figure 5-figure supplement 1 - source data 2.tif]
